# 72 Burn Rehabilitation Non-invasive Approach; Lower Extremity Burns

**DOI:** 10.1093/jbcr/irac012.075

**Published:** 2022-03-23

**Authors:** David H Funk, Sandra Fletchall, Sai R Velamuri

**Affiliations:** Firefighter's Burn Center, Memphis, Tennessee; Firefighter's Burn Center, Memphis, Tennessee; Firefighter's Burn Center, Memphis, Tennessee

## Abstract

**Introduction:**

An increase intolerance to weight-bearing or ambulation was noted when second degree burns were sustained in the lower extremities, specifically knee distally. Patients were noted to request additional pain medication and/or additional time in the bed, which are not this burn center’s burn rehabilitation activity protocol.

Use of double ace wraps, from knee distally, was initiated to increase patient’s participation in burn rehabilitation therapy. A retrospective chart review was used to determine if there were similar factors could be identified to anticipate use of a lower extremity double ace wrap.

**Methods:**

Retrospective chart review from July 2020 to August 2021 was performed to identify lower extremity burns. Inclusion criteria included: age 13 or greater; admitted to this burn center w/LOS greater than 24 hours, received burn rehabilitation therapy; second and third degree burns located below the knee; and pre-admission status of independent ambulator.

Chart review included 104 patients, with criteria met by 19 males and 5 females. The average age was 38.9 years with a range of 13 to 78 years. Length of stay ranged from 1 to 30 days and pre-exiting medical history included diabetes, type II in 8.3% and hypertension in 16.7% of the patients.

The TBSA ranged from 1.15% to 42%. Surgical procedures of BWE w/dermal/epidermal skin substitute application from knee distally, occurred in 50% of the patients; 37.5% underwent BWE w/allograft and within 5-7 days underwent meshed split thickness skin grafts; 4% underwent BWE with placement of xeroform. 8.3% did not require surgery.

**Results:**

All patients involved in the study required use of double ace wraps during hospital stay. Duration of use of double ace wraps depended on subjective report of abnormal sensations. Use was required mainly in the acute phase of burn injury prior to autografting, in partial thickness burns that underwent surgical procedure for application of dermal/epidermal skin substitute, or in patients that did not require autografting.

Patient’s verbalizations of abnormal sensations with attempts to immediately weight bear or ambulate included: shooting pain, stinging pain, throbbing sensation, pins and needles. Reports of abnormal sensations and increased compliance with burn rehabilitation therapy was noted immediately following application of double ace wrap.

**Conclusions:**

Burn Rehabilitation therapy protocol now includes close identification of superficial or deep second degrees located distally to the knee and patient’s verbal descriptions of abnormal sensations with attempts to place feet into a dependent position. Burn Rehabilitation therapy has developed a wearing schedule of the double ace wrap which has resulted in no adverse effects and positive patient compliance and participation.

